# Patients with preoperative bone marrow oedema benefit more substantially from medial meniscus posterior root repair

**DOI:** 10.1002/ksa.12792

**Published:** 2025-07-18

**Authors:** Junwoo Byun, Sung‐Hwan Kim, Chong‐Hyuk Choi, Min Jung, Kwangho Chung, Se‐Han Jung, Jun‐Young Lim, Hyun‐Soo Moon

**Affiliations:** ^1^ Arthroscopy and Joint Research Institute, Yonsei University College of Medicine Seoul Republic of Korea; ^2^ Department of Orthopaedic Surgery, Mokdong Hospital Ewha Womans University College of Medicine Seoul Republic of Korea; ^3^ Department of Orthopedic Surgery, Severance Hospital Yonsei University College of Medicine Seoul Republic of Korea; ^4^ Department of Orthopedic Surgery Yonsei Bonsarang Hospital Bucheon Republic of Korea; ^5^ Department of Orthopedic Surgery, Yongin Severance Hospital Yonsei University College of Medicine Yongin Republic of Korea; ^6^ Department of Orthopedic Surgery, Gangnam Severance Hospital Yonsei University College of Medicine Seoul Republic of Korea

**Keywords:** bone marrow oedema, functional improvement, medial meniscus root tear, surgical outcome

## Abstract

**Purpose:**

To analyze the clinical outcomes of the surgical repair of medial meniscus posterior root tear (MMRT) in patients with preoperative bone marrow oedema (BME).

**Methods:**

Patients who underwent arthroscopic pull‐out repair for MMRT between 2010 and 2022 were retrospectively reviewed, and those with a minimum of two years of follow‐up were included. The patients were then classified into two groups based on the presence of BME in the medial compartment of the knee on preoperative magnetic resonance imaging (Group 1: patients without BME, Group 2: patients with BME). A comparative analysis was conducted for subjective and objective outcomes, including clinical scores and radiological parameters, at 2 years post‐operatively and at the final follow‐up. In particular, the minimal clinically important difference (MCID), substantial clinical benefit (SCB), and patient acceptable symptom state (PASS) values for both the International Knee Documentation Committee (IKDC) subjective and Lysholm scores were used to evaluate post‐operative clinical improvement compared to preoperative baseline scores.

**Results:**

A total of 95 patients were included (Group 1: 54 patients, mean follow‐up 4.1 ± 2.1 years; Group 2: 41 patients, mean follow‐up 4.2 ± 2.3 years). There were no significant differences in demographic and preoperative data, radiological parameters, or clinical scores at 2 years post‐operatively or at the final follow‐up. Similar results were observed consistently across subgroup analyses stratified by BME severity. However, the proportion of patients who achieved PASS, improvement beyond MCID at 2 years post‐operatively, and SCB at the final follow‐up for IKDC subjective score was significantly higher in Group 2 (*p* = 0.049, *p* = 0.047 and *p* = 0.038, respectively).

**Conclusions:**

Preoperative BME in patients undergoing surgical repair for MMRT did not appear to affect short‐ and mid‐term outcomes but was indeed associated with a higher likelihood of functional improvement. Thus, preoperative BME need not be a concern in the surgical repair of MMRT.

**Level of Evidence:**

Level III.

**Abbreviations**
BME: bone marrow oedema
HKA: hip–knee–ankle
ICC: intraclass correlation coefficient
ICRS: International Cartilage Research Society
IKDC: International Knee Documentation Committee
MCID: minimal clinically important difference
MME: medial meniscus extrusion
MMRT: medial meniscus posterior root tear
MRI: magnetic resonance imaging
PASS: patient acceptable symptom state
SCB: substantial clinical benefit
ΔMME: changes in MME between these two time points

## INTRODUCTION

Medial meniscus posterior root tear (MMRT) compromises the intrinsic function of the medial meniscus, and surgical repair is generally recommended unless specific contraindications exist [15, 18, 28, 29, 39, 43]. Although surgical repair for MMRT does not fully restore meniscal function or entirely halt the progression of osteoarthritis, it has consistently been reported to offer significant clinical advantages over nonoperative treatment or meniscectomy [5, 10, 23, 25, 30, 33, 34, 38]. Consequently, the frequency of surgical repair for MMRT has been steadily increasing and is currently regarded as the standard treatment option for this condition [11].

Notably, bone marrow oedema (BME) in the medial compartment of the knee is frequently observed on magnetic resonance imaging (MRI) at the time of MMRT diagnosis, with a reported incidence of 33.3% [3]. BME is commonly associated with degenerative changes in the knee joint and has been reported to contribute to subjective pain and osteoarthritis progression [6, 8, 9, 26, 37]. This radiologic finding is believed to result from increased stress on the articular cartilage, which may be caused by the loss of meniscal function in patients with MMRT [1, 12]. Given its characteristics, it is logical to assume that the presence of preoperative BME could adversely affect clinical outcomes following surgical repair of MMRT. Nevertheless, there is a paucity of studies specifically analyzing the impact of this effect. Evaluating clinical outcomes based on the presence of preoperative BME in patients undergoing MMRT could provide valuable prognostic insights and help establish clinical guidelines for treatment decision‐making, particularly when determining whether surgical repair should be pursued.

Therefore, this study aimed to analyze the clinical outcomes of surgical repair of MMRT in patients with preoperative BME. We hypothesized that the presence of preoperative BME would result in inferior clinical outcomes compared to those without BME.

## METHODS

**Figure 1:**
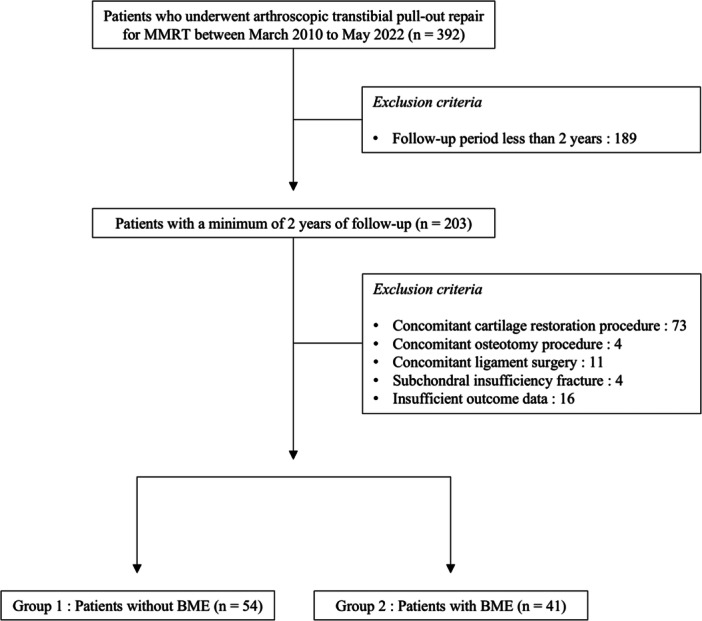
Flowchart of patient inclusion in the study. BME, bone marrow oedema; MMRT, medial meniscus posterior root tear.

The medical records of patients who underwent surgical repair of MMRT by two senior surgeons at a single institution between March 2010 and May 2022 were retrospectively reviewed. Patients meeting any of the following criteria were excluded from the analysis: (1) follow‐up period less than 2 years, (2) concomitant cartilage restoration procedure, (3) concomitant osteotomy procedure, (4) concomitant ligament surgery, (5) subchondral insufficiency fracture of the affected knee or (6) insufficient outcome data. The distinction of subchondral insufficiency fracture of the knee from BME was made based on the presence of a subchondral hypointense line on the MRI [35]. Eligible patients were then categorized into two groups based on the presence or absence of BME observed on MRI at the time of MMRT diagnosis in the medial compartment of the affected knee (Group 1, patients without BME; Group 2, patients with BME) (Figure 1).

### Surgical indication, technique and post‐operative rehabilitation

The indications for the surgical repair of MMRT at our institution required patients to meet all of the following criteria: nonelderly patients (typically ≤65 years of age), radiographic osteoarthritis graded ≤2 according to the Kellgren–Lawrence grading scale, absence of pathological varus alignment in the lower extremity (hip–knee–ankle [HKA] angle ≤10° and <5° compared to the contralateral limb on radiographs), and a willingness to adhere to a strict post‐operative rehabilitation programme. In cases where cartilage lesions were present in the medial compartment of the knee, their severity was intraoperatively assessed through arthroscopic examination using the International Cartilage Research Society (ICRS) grading system, and cartilage restoration procedures were performed for lesions graded >3a.

The surgical procedure was performed using the arthroscopic transtibial pull‐out repair technique. A modified reverse Mason‐Allen stitch, comprising a horizontal loop made with an ultra‐high‐molecular‐weight polyethylene suture and an overlaid vertical locking component using a No. 1 polydioxanone suture, was applied [30]. The stitch was placed approximately 3–5 mm away from the torn edge of the medial meniscus posterior root. A tibial tunnel was created under direct visualization through the posteromedial portal, and sutures were secured over the anteromedial tibial cortex using Endobutton (Smith & Nephew) [31]. After surgery, the patients were instructed to use crutches and a hinged knee brace, maintaining a toe‐touch weight‐bearing gait for the first 4 weeks, followed by partial weight‐bearing for the next 6 weeks. Throughout the toe‐touch and partial weight‐bearing periods, crutch‐assisted ambulation was performed exclusively with the knee fully extended. Passive range of motion exercises were initiated 2 weeks post‐operatively, with active range of motion exercises and unrestricted full weight‐bearing permitted after 10 weeks.

### Patient evaluation

Demographic and intraoperative data, clinical scores and radiological parameters were analyzed retrospectively. Intraoperative data included the cartilage status of the medial tibiofemoral joint, documented immediately after surgery using the ICRS grading system. If cartilage lesions were present on both the femoral condyle and tibial plateau, the lesion with a higher grade was used for evaluation. Subjective clinical outcomes were assessed using the International Knee Documentation Committee (IKDC) subjective and the Lysholm scores, with data obtained preoperatively, 2 years post‐operatively, and at the final follow‐up. Established thresholds reported by previous studies for the minimal clinically important difference (MCID), substantial clinical benefit (SCB) and patient acceptable symptom state (PASS) were applied to determine clinically meaningful improvements after surgery for each score (11.5, 30.6 and 69.0, respectively, for the IKDC subjective score, and 10.1, 24.5 and 61.0, respectively, for the Lysholm score) [2, 14, 27, 41].

**Figure 2:**
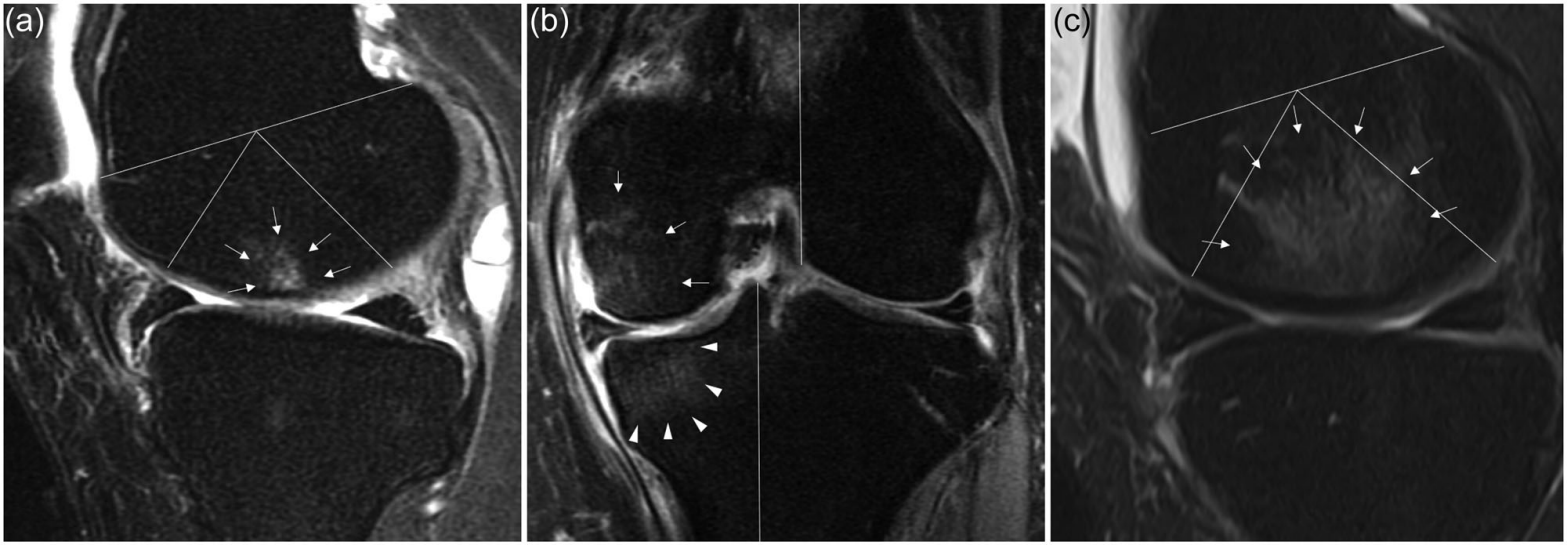
BME severity assessment based on the extent of its anatomically defined subregional involvement observed on MRI. (a) Sagittal T2 fat suppression image with Grade 1 BME depicted on the central subregion of medial femur (arrow). (b) Coronal T2 fat suppression image with Grade 2 BME depicted on medial tibia subregion (arrowhead) and Grade 1 BME on central subregion of medial femur (arrow). (c) Sagittal T2 fat suppression image with Grade 3 BME depicted on central subregion of medial femur. BME, bone marrow oedema; MRI, magnetic resonance imaging.

The radiological parameters utilized in this study to evaluate patients included the radiographic osteoarthritis grade and HKA angle, both measured on plain radiographs, as well as the medial meniscus extrusion (MME), the healing status of the repaired MMRT, the location of the intra‐articular transosseous tibial tunnel, and the presence and characteristics of BME, all assessed using MRI. On standing anteroposterior radiographs of the knee, the osteoarthritis grade was evaluated based on the Kellgren–Lawrence grading system. The HKA angle was determined using full‐length weight‐bearing anteroposterior radiographs of the lower extremities. These parameters were assessed using imaging data obtained preoperatively, at 2 years post‐operatively, and at the final follow‐up. The MME was assessed on the coronal plane of the MRI following the method outlined by Costa et al. [32]. MME measurements were performed using MRI scans obtained preoperatively and 1 year post‐operatively. The healing status of the repaired MMRT was evaluated based on the approach described by Kim et al., which involved assessing the continuity between the repaired MMRT and the bone in the coronal, sagittal and axial planes [19]. The location of the intraarticular transosseous tibial tunnel was examined in the axial plane. The tunnel was deemed anatomically positioned if it was located within 0.8 cm of the medial edge of the posterior cruciate ligament at the level of the joint surface [16]. BME was defined as an ill‐delineated bone marrow lesion adjacent to the articular surface on the medial compartment of the affected knee, characterized by a hypointense signal on T1‐weighted images and a hyperintense signal on T2‐weighted images observed on MRI [42]. Lesions with well‐defined borders, which are more likely indicative of osteonecrosis, or those not located adjacent to the subchondral area, which are presumed to result from other etiologies, were not classified as BME [1]. The severity of BME was classified based on the extent of its involvement [13]. The extent of BME involvement was graded by dividing the femur and tibia into anatomically defined subregions on the sagittal and coronal MRI images. The femur was divided into the trochlea, central and posterior regions on the sagittal plane, while the tibia was divided into anterior, central and posterior subregions. In the coronal plane, the tibia was categorized into the medial, subspinous and lateral regions, and the femur was categorized into the medial and lateral femoral condyles, with the intercondylar notch included in the medial condyle. Based on these subregional divisions, the severity of BME was graded according to the percentage of subregional volume affected (Grade 0 = no involvement, Grade 1 ≤ 33% of the subregional volume, Grade 2 = 33%–66% and Grade 3 ≥ 66%) (Figure 2). To analyze the effect of BME severity on post‐operative outcomes, a subgroup analysis was performed by comparing groups classified according to the highest BME grade among the subregions.

The analysis of radiologic parameters was performed by an experienced orthopaedic surgeon who was blinded to patient information. Two separate measurements were conducted at 2‐week intervals, and the previous values were concealed during the second measurement. Continuous variables were evaluated by calculating the average of two independent measurements. For categorical variables, discrepancies between the two measurements were resolved through consultation with the senior author.

### Ethical approval

This study was approved by the Institutional Review Board of Severance Hospital, and the requirement for informed consent was waived owing to its retrospective nature (IRB number 4‐2024‐1164).

### Statistical analysis

Prior to the study, a priori power analysis was performed to determine the minimum sample size required to detect differences in surgical outcomes between the groups. Variables included in the power analysis were the IKDC subjective score, Lysholm score and MME. Clinically established thresholds (IKDC subjective score, with an MCID set at 11.5; Lysholm score, with an MCID set at 10.1; and MME, indicating a clinically relevant change of 1.6 mm) and standard deviation values from prior studies served as reference points for the calculations [2, 4, 14, 20]. Based on this analysis, with an alpha level of 5% and a statistical power of 90%, it was estimated that a minimum of 17 patients per group would be necessary for a valid comparison of surgical outcomes.

For comparisons between groups, continuous variables were analyzed using Student's *t* test for normally distributed data and the Mann–Whitney *U* test for non‐normally distributed data. Categorical variables were compared using Pearson's chi‐square test or Fisher's exact test. Additionally, to evaluate the impact of BME severity on surgical outcomes, one‐way analysis of variance using Scheffé's post‐hoc test was employed for multiple group comparisons of continuous variables, whereas Pearson's chi‐square test or Fisher's exact test was applied for categorical variables. To assess the reliability of the radiological measurements, intraclass correlation coefficients (ICCs) with a 95% confidence interval were calculated for continuous variables, whereas weighted kappa coefficients were used for categorical variables. The level of statistical significance was set at *p* < 0.05.

## RESULTS

**Figure 3:**
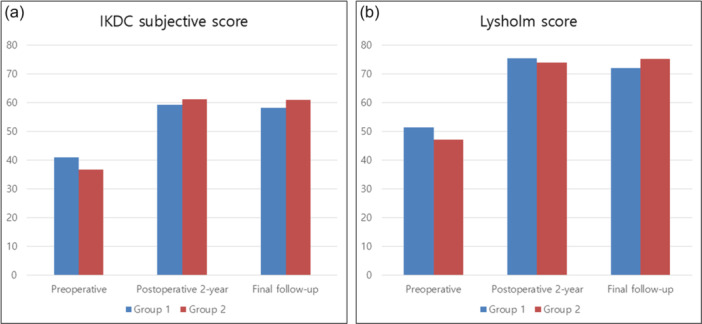
Between‐group comparison of (a) IKDC subjective score and (b) Lysholm score. IKDC, International Knee Documentation Committee.

A total of 95 patients were included in this study, with 54 in Group 1 and 41 in Group 2 (Figure 1). No significant differences were observed in the demographic characteristics, intraoperative data, radiologic data and clinical scores at each time point between the two groups (Tables 1, 2, 3, Figure 3). However, the proportion of patients achieving PASS and clinical improvement beyond the MCID at 2 years post‐operatively, as well as SCB at the final follow‐up, for the IKDC subjective score was significantly higher in Group 2 (*p* = 0.049, 0.047 and 0.038, respectively) (Table 3).

**Table 1:** Comparison of baseline demographic data and intraoperative data.

Variables^a^	Group 1^b^ (*n* = 54)	Group 2^b^ (*n* = 41)	*p* value
Demographic data			
Age, years	57.7 ± 8.6	58.7 ± 6.7	n.s.
Sex, male/female^c^	9/45	5/36	n.s.
Body mass index, kg/m^2^	25.9 ± 3.4	26.9 ± 3.3	n.s.
Affected side, right/left c	20/34	16/25	n.s.
Preoperative symptom duration, weeks^d^	20.1 ± 18.3	15.0 ± 13.1	n.s.
Follow‐up MRI, yes/no^c^	26/28	21/20	n.s.
Follow‐up duration, years	4.1 ± 2.1	4.2 ± 2.3	n.s.
Intraoperative data			
ICRS grade of cartilage lesion, 0/1/2/3a^c^	15/2/16/21	5/5/15/16	n.s.

Abbreviations: BME, bone marrow oedema; ICRS, International Cartilage Research Society; MRI, magnetic resonance imaging.

^a^ The values are given as the mean and standard deviation, otherwise noted separately.

^b^ Group 1, patients without BME; Group 2, patients with BME.

^c^ The values are given as number of patients.

^d^ One patient and two patients in Groups 1 and 2, respectively, were excluded from the analysis due to the lack of relevant information.

**Table 2:** Comparison of radiographic parameters.

Variables^a^	Group 1^b^ (*n* = 54)	Group 2^b^ (*n* = 41)	*p* value
Kellgren–Lawrence grade^c^			
Preoperative, 0/1/2	17/33/4	12/27/2	n.s.
Post‐operative 2 years, 0/1/2/3	4/28/16/6	1/24/15/1	n.s.
Progression of Kellgren–Lawrence grade, yes/no^d^	29/25	24/17	n.s.
Final follow‐up, 0/1/2/3/4	3/27/15/9/0	1/19/15/5/1	n.s.
Progression of Kellgren–Lawrence grade, yes/no^d^	31/23	27/14	n.s.
HKA angle,°			
Preoperative	3.7 ± 2.4	3.2 ± 3.0	n.s.
Post‐operative 2 years	4.6 ± 2.6	4.1 ± 3.3	n.s.
Δ HKA angle,°^d^	0.9 ± 1.1	0.8 ± 1.2	n.s.
Final follow‐up	4.6 ± 2.7	4.5 ± 3.4	n.s.
Δ HKA angle,°^d^	0.9 ± 1.3	1.2 ± 1.4	n.s.
Meniscus extrusion, mm			
Preoperative	2.5 ± 0.8	2.7 ± 0.9	n.s.
Post‐operative 1 year^e^	3.8 ± 1.3	3.6 ± 1.4	n.s.
Δ Meniscus extrusion, mm^d^^,^ e	1.3 ± 1.3	0.8 ± 1.0	n.s.
Healing of MMRT, complete/partial/no^c^^,^ e	18/6/2	14/7/0	n.s.
Tunnel position, anatomic/non‐anatomic^c^^,^ e	22/4	16/5	n.s.
Post‐operative BME, yes/no^c^^,^ e	7/19	10/11	n.s.

Abbreviations: Δ, The difference of value between two time points; BME, bone marrow oedema; HKA, hip–knee–ankle; MMRT, medial meniscus posterior root tear.

^a^ The values are given as the mean and standard deviation, otherwise noted separately.

^b^ Group 1, patients without BME; Group 2, patients with BME.

^c^ The values are given as number of patients.

^d^ Comparison with the corresponding preoperative variables.

^e^ Analysis of patients who underwent follow‐up MRI.

**Table 3:** Comparison of clinical scores.

Variables^a^	Group 1^b^ (*n* = 54)	Group 2^b^ (*n* = 41)	*p* value
IKDC subjective score			
Preoperative^c^	41.0 ± 14.8	36.7 ± 15.2	n.s.
Post‐operative 2 years^c^	59.3 ± 15.3	61.2 ± 17.2	n.s.
Clinical improvement beyond the MCID, yes/no^d^	33/21	33/8	0.047
Clinical improvement beyond the SCB, yes/no^d^	28/26	25/16	n.s.
Clinical outcome beyond the PASS, yes/no	13/41	18/23	0.049
Final follow‐up^c^	58.2 ± 16.7	61.0 ± 19.3	n.s.
Clinical improvement beyond the MCID, yes/no^d^	35/19	29/12	n.s.
Clinical improvement beyond the SCB, yes/no^d^	22/32	26/15	0.038
Clinical outcome beyond the PASS, yes/no	15/39	16/25	n.s.
Lysholm score			
Preoperative^c^	51.3 ± 21.7	47.1 ± 23.3	n.s.
Post‐operative 2 years^c^	75.5 ± 18.4	74.0 ± 20.1	n.s.
Clinical improvement beyond the MCID, yes/no^d^	36/18	31/10	n.s.
Clinical improvement beyond the SCB, yes/no^d^	32/22	26/15	n.s.
Clinical outcome beyond the PASS, yes/no	26/28	18/23	n.s.
Final follow‐up^c^	72.0 ± 21.1	75.2 ± 23.2	n.s.
Clinical improvement beyond the MCID, yes/no^d^	35/19	33/8	n.s.
Clinical improvement beyond the SCB, yes/no^d^	29/25	26/15	n.s.
Clinical outcome beyond the PASS, yes/no	21/33	20/21	n.s.

Abbreviations: IKDC, International Knee Documentation Committee; MCID, minimal clinically important difference; PASS, patient acceptable symptom state; SCB, substantial clinical benefit.

^a^ The values are given asthe number of patients, otherwise noted separately.

^b^ Group 1, patients without BME; Group 2, patients with BME.

^c^ The values are given as the mean and standard deviation.

^d^ Comparison of the corresponding preoperative values

In addition to comparison between the two groups, subgroup analyses were performed to assess the influence of preoperative BME severity on clinical outcomes. Group 2 was subdivided into three subgroups based on preoperative BME grading. The results showed no significant differences in demographic characteristics, intraoperative data, radiological parameters, or clinical scores among the groups, indicating that preoperative BME severity also had no significant impact on clinical outcomes (Supportig Information S1: Tables 1–4).

The reliability of the radiological measurements for continuous variables was evaluated as good to excellent, with ICC values ranging from 0.861 to 0.996 [22]. For categorical variables, reliability was assessed as having substantial agreement, with kappa values between 0.632 and 0.785 [24].

## DISCUSSION

The primary finding of this study was that preoperative BME did not adversely affect surgical outcomes following MMRT repair. This result contradicted our initial hypothesis, as it revealed that patients with preoperative BME were more likely to achieve significant clinical improvement than those without preoperative BME.

BME, a nonspecific MRI finding, can stem from multiple etiologies. Although commonly observed in association with osteoarthritis, it is also linked to various other pathological conditions of the joint [21, 37, 40, 42]. In particular, BME observed alongside a meniscal tear is likely attributable to the increased mechanical stress applied to the joint. Such stress can alter the loading condition in the knee joint, increase the stress on the subchondral bone and promote the development of BME [21, 40, 42]. Additionally, meniscal tears may compromise joint stability and biomechanics, further contributing to the progression of BME [7, 36]. Notably, these processes may theoretically accelerate degenerative changes in the joint and could also correlate with the patient's subjective symptoms. Given these considerations, surgeons may be reasonably concerned about the prognosis when BME is present in patients undergoing surgical repair for MMRT. Furthermore, they might question whether surgical repair is an appropriate treatment option for MMRT in such cases. Nevertheless, few studies have examined the impact of preoperative BME on the surgical outcomes of MMRT repair. Therefore, this study aimed to investigate the relationship between preoperative BME and the clinical outcomes of surgical repair of MMRT.

This study demonstrated that there were no differences in surgical outcomes, including clinical scores and radiological parameters, between groups based on the presence of preoperative BME. Furthermore, these findings were consistent across subgroup comparisons based on the BME severity. Interestingly, patients with preoperative BME were more likely to achieve post‐operative clinical improvement, as observed in the IKDC subjective score at both the 2‐year follow‐up and final evaluation. Although the differences were not statistically significant, patients with preoperative BME tended to exhibit lower clinical scores preoperatively and higher scores post‐operatively than those without BME. This pattern suggests that more severe preoperative symptoms in patients with BME may have contributed to greater perceived post‐operative improvement, leading to higher satisfaction levels. Overall, preoperative BME does not appear to adversely affect clinical outcomes following surgical repair of MMRT.

Notably, while differences in the presence of preoperative BME were observed between the groups, no significant differences were observed in the proportion of patients with BME at 1‐year follow‐up. This indicates that some patients with preoperative BME showed resolution, whereas others without BME developed it over time. Chung et al. have similarly reported that BME may either improve or worsen after surgical repair of MMRT, potentially influencing clinical outcomes [3]. Given the multifactorial nature of BME, interpretation of these findings remains challenging. The disappearance of BME might signify the restoration of normal loading conditions in the knee following surgical repair. Conversely, persistent or newly developed BME can indicate inadequate functional recovery or continued biomechanical abnormalities [1, 7, 12]. Furthermore, the emergence or persistence of BME could occur independently as a secondary phenomenon due to ongoing degenerative changes in the knee joint [9, 26]. What is critical is that preoperative BME associated with MMRT surgical repair shows potential for resolution, which may align with improved clinical outcomes. Consequently, surgeons should actively consider surgical repair for MMRT in patients with BME, rather than hesitating due to its presence.

The clinical outcomes of surgical repair of MMRT have not yet been considered fully satisfactory [17, 30]. Accordingly, when BME, a condition associated with both subjective symptoms and degenerative changes in the joint, is observed along with MMRT, surgeons are likely to be more concerned with the outcomes of MMRT repair. They may even question whether surgical repair is an appropriate treatment option in such cases. Encouragingly, this study revealed that BME associated with MMRT does not negatively impact surgical outcomes and may even contribute positively to patient‐reported satisfaction. Therefore, when preparing for surgical repair of MMRT, surgeons should pay less attention to the presence of frequently observed BME and focus instead on minimizing its persistence or emergence post‐operatively.

This study has several limitations. First, because this was a retrospective study, there may have been a risk of bias during evaluation. Second, the number of patients included in this study was relatively small. Although the sample size met the requirements determined by the power analysis, the conclusions were based on representative variables and may not be applicable to all variables. Third, not all patients underwent follow‐up MRI, which was further limited to evaluations only at the 1‐year post‐operative time point, potentially leading to a relatively insufficient assessment of MRI‐related radiological parameters.

## CONCLUSIONS

Preoperative BME in patients undergoing surgical repair for MMRT did not appear to affect short‐ and mid‐term outcomes but was indeed associated with a higher likelihood of functional improvement. Thus, preoperative BME need not be a concern in the surgical repair of MMRT.

## AUTHOR CONTRIBUTIONS

The project was coordinated by Junwoo Byun and Hyun‐Soo Moon. Junwoo Byun drafted the manuscript, together with Kwangho Chung and Se‐Han Jung. Junwoo Byun and Hyun‐Soo Moon generated the concept of the study. The acquisition of data and analysis was done by Se‐Han Jung and Jun‐Young Lim. Min Jung, Sung‐Hwan Kim and Chong‐Hyuk Choi provided supervision and guidance throughout the research process. The design of the study and the interpretation of data were all done jointly by all authors. Junwoo Byun and Hyun‐Soo Moon revised the final draft critically for important intellectual content and approved the version to be submitted. All of the authors agreed to be accountable for all aspects of the work to ensure that questions related to the accuracy or integrity of any part of the work are appropriately investigated and resolved.

## CONFLICT OF INTEREST STATEMENT

The authors declare no conflicts of interest.

## ETHICS STATEMENT

This study was ethically approved by the Institutional Review Board of Severance Hospital.

## Supporting information

Supplementary Information

## Data Availability

The data that support the findings of this study are available from the corresponding author upon reasonable request.
