# Supplementary material for: Patients with preoperative bone marrow oedema benefit more substantially from medial meniscus posterior root repair
Source: Knee Surg Sports Traumatol Arthrosc. 2025 Jul 18;34(3):834–43. doi: 10.1002/ksa.12792 (PMC12948345; doi:10.1002/ksa.12792)
Supplement: Supplementary file 1 — Supplementary Information [file KSA-34-834-s001.docx]

**Supplementary Table 1** Subgroup Analysis Comparing Baseline Demographic Data and Intraoperative Data

| Variables*^a^* | Group 1*^b^*  (n = 54) | BME 1*^b^*  (n = 16) | BME 2*^b^*  (n = 6) | BME 3*^b^*  (n = 19) | *P* value |
| --- | --- | --- | --- | --- | --- |
| Demographic data |  |  |  |  |  |
| Age, years | 57.7 ± 8.6 | 58.5 ± 7.0 | 61.2 ± 9.1 | 58.0 ± 5.7 | n.s. |
| Sex, male/female*^c^* | 9/ 45 | 1/ 15 | 0/ 6 | 4/ 15 | n.s. |
| Body mass index, kg/m^2^ | 25.9 ± 3.4 | 27.2 ± 2.9 | 25.1 ± 2.8 | 27.2 ± 3.8 | n.s. |
| Affected side, right/ left*^c^* | 20/ 34 | 6/ 10 | 2/ 4 | 8/ 11 | n.s. |
| Preoperative symptom duration, weeks*^d^* | 20.1 ± 18.3 | 18.1 ± 18.0 | 12.5 ± 8.8 | 13.5 ± 9.7 | n.s. |
| Follow-up MRI, yes/ no*^c^* | 26/ 28 | 5/ 11 | 4/ 2 | 12/ 7 | n.s. |
| Follow-up duration, years | 4.1 ± 2.1 | 4.8 ± 2.6 | 3.8 ± 1.0 | 4.0 ± 2.3 | n.s. |
| Intraoperative data |  |  |  |  |  |
| ICRS grade of cartilage lesion, 0/1/2/3a*^c^* | 15/ 2/ 16/ 21 | 1/ 3/ 5/ 7 | 1/ 0/ 2/ 3 | 3/ 2/ 8/ 6 | n.s. |

BME, Bone marrow edema; MRI, Magnetic resonance imaging; ICRS, International Cartilage Research Society

*^a^* The values are given as the mean and standard deviation, otherwise noted separately

*^b^* Group 1, patients without BME; BME 1, patients with a maximum BME grade of 1; BME 2, patients with a maximum BME grade of 2; BME 3, patients with a maximum BME grade of 3.

*^c^* The values are given as number of patients

*^d^* One patient and two patients in group 1 and BME 1 respectively were excluded from the analysis due to the lack of relevant information

**Supplementary Table** **2** Subgroup Analysis Comparing Radiologic Parameters

| Variables*^a^* | Group 1*^b^*  (n = 54) | BME 1*^b^*  (n = 16) | BME 2*^b^*  (n = 6) | BME 3*^b^*  (n = 19) | *P* value |
| --- | --- | --- | --- | --- | --- |
| Kellgren-Lawrence grade*^c^* |  |  |  |  |  |
| Preoperative, 0/ 1/ 2 | 17/ 33/ 4 | 1/ 14/ 1 | 3/ 3/ 0 | 8/ 10/ 1 | n.s. |
| Postoperative 2-year, 0/ 1/ 2/ 3 | 4/ 28/ 16/ 6 | 0/ 8/ 7/ 1 | 0/ 4/ 2/ 0 | 1/ 12/ 6/ 0 | n.s. |
| Progression of Kellgren-Lawrence grade, yes/ no*^e^* | 29/ 25 | 9/ 7 | 4/ 2 | 11/ 8 | n.s. |
| Final follow-up, 0/ 1/ 2/ 3/ 4 | 3/ 27/ 15/ 9/ 0 | 0/ 8/ 5/ 2/ 1 | 0/ 2/ 2/ 2/ 0 | 1/ 9/ 8/ 1 /0 | n.s. |
| Progression of Kellgren-Lawrence grade, yes/ no*^e^* | 31/ 23 | 9/ 7 | 5/ 1 | 13/ 6 | n.s. |
| HKA angle, ° |  |  |  |  |  |
| Preoperative | 3.7 ± 2.4 | 1.8 ± 3.2 | 5.1 ± 3.0 | 3.9 ± 2.5 | 0.027 |
| Postoperative 2-year | 4.6 ± 2.6 | 2.5 ± 3.1 | 6.0 ± 4.3 | 4.8 ± 2.7 | 0.026 |
| Δ HKA angle, °*^e^* | 0.9 ± 1.1 | 0.7 ± 1.0 | 0.9 ± 2.1 | 0.9 ± 1.0 | n.s. |
| Final follow-up | 4.6 ± 2.7 | 3.0 ± 3.4 | 6.7 ± 4.4 | 5.1 ± 2.5 | 0.036 |
| Δ HKA angle, °*^e^* | 0.9 ± 1.3 | 1.1 ± 1.4 | 1.6 ± 2.6 | 1.2 ± 0.9 | n.s. |
| Meniscus Extrusion, mm |  |  |  |  |  |
| Preoperative, mm | 2.5 ± 0.8 | 2.4 ± 1.1 | 2.9 ± 0.6 | 2.9 ± 0.9 | n.s. |
| Postoperative 1-year, mm*^d^* | 3.8 ± 1.3 | 4.1 ± 1.7 | 3.8 ± 1.0 | 3.3 ± 1.4 | n.s. |
| Δ Meniscus Extrusion, mm*^de^* | 1.3 ± 1.3 | 1.3 ± 1.1 | 1.0 ± 0.9 | 0.5 ± 1.0 |  |
| Healing of MMRT, complete/ partial/ no*^cd^* | 18/ 6/ 2 | 5/ 0/ 0 | 3/ 1/ 0 | 6/ 6/ 0 | n.s. |
| Tunnel position, anatomic/ nonanatomic*^cd^* | 22/ 4 | 4/ 1 | 3/ 1 | 9/ 3 | n.s. |
| Postoperative BME, yes/ no*^cd^* | 7/ 19 | 2/ 3 | 2 /2 | 6/ 6 | n.s. |

BME, bone marrow edema; HKA, Hip-Knee-Ankle; Δ, The difference of value between two time points; MMRT, Medial meniscus posterior root tear

*^a^* The values are given as the mean and standard deviation, otherwise noted separately

*^b^* Group 1, patients without BME; BME 1, patients with a maximum BME grade of 1; BME 2, patients with a maximum BME grade of 2; BME 3, patients with a maximum BME grade of 3.

*^c^* The values are given as number of patients

*^d^* Analysis of patients who underwent follow-up MRI

*^e^* Comparison with the corresponding preoperative variables

**Supplementary Table 3** Subgroup Analysis Comparing Clinical Scores

| Variables*^a^* | Group 1*^b^*  (n = 54) | BME 1*^b^*  (n = 16) | BME 2*^b^*  (n = 6) | BME 3*^b^*  (n = 19) | *P* value |
| --- | --- | --- | --- | --- | --- |
| IKDC subjective score |  |  |  |  |  |
| Preoperative*^c^* | 41.0 ± 14.8 | 41.5 ± 14.7 | 33.0 ± 15.8 | 33.9 ± 15.1 | n.s. |
| Postoperative 2-years*^c^* | 59.3 ± 15.3 | 60.4 ± 18.0 | 61.3 ± 15.9 | 61.8 ± 17.7 | n.s. |
| Clinical improvement beyond the MCID, yes/ no *^d^* | 33/ 21 | 14/ 2 | 4/ 2 | 15/ 4 | n.s. |
| Clinical improvement beyond the SCB, yes/ no *^d^* | 28/ 26 | 10/ 6 | 4/ 2 | 11/ 8 | n.s. |
| Clinical outcome beyond the PASS, yes/ no | 30/ 24 | 7/ 9 | 3/ 3 | 10/ 9 | n.s. |
| Final follow-up*^c^* | 58.2 ± 16.7 | 58.6 ± 20.1 | 59.2 ± 24.7 | 63.5 ± 17.4 | n.s. |
| Clinical improvement beyond the MCID, yes/ no *^d^* | 35/ 19 | 11/ 5 | 3/ 3 | 15/ 4 | n.s. |
| Clinical improvement beyond the SCB, yes/ no *^d^* | 22/ 32 | 10/ 6 | 3/ 3 | 13/ 6 | n.s. |
| Clinical outcome beyond the PASS, yes/ no | 25/ 29 | 8/ 8 | 3/ 3 | 12/ 7 | n.s. |
| IKDC subjective score |  |  |  |  |  |
| Preoperative*^c^* | 51.3 ± 21.7 | 52.9 ± 22.7 | 48.3 ± 20.8 | 41.7 ± 24.3 | n.s. |
| Postoperative 2-years*^c^* | 75.5 ± 18.4 | 74.9 ± 20.0 | 75.2 ± 20.2 | 72.8 ± 21.1 | n.s. |
| Clinical improvement beyond the MCID, yes/ no *^d^* | 36/ 18 | 13/ 3 | 5/ 1 | 13/ 6 | n.s. |
| Clinical improvement beyond the SCB, yes/ no *^d^* | 32/ 22 | 9/ 7 | 5/ 1 | 12/ 7 | n.s. |
| Clinical outcome beyond the PASS, yes/ no | 26/ 28 | 7/ 9 | 2/ 4 | 9/ 10 | n.s. |
| Final follow-up*^c^* | 72.0 ± 21.1 | 75.8 ± 23.5 | 75.8 ± 30.8 | 74.5 ± 21.7 | n.s. |
| Clinical improvement beyond the MCID, yes/ no *^d^* | 35/ 19 | 13/ 3 | 4/ 2 | 16/ 3 | n.s. |
| Clinical improvement beyond the SCB, yes/ no *^d^* | 29/ 25 | 9/ 7 | 4/ 2 | 13/ 6 | n.s. |
| Clinical outcome beyond the PASS, yes/ no | 21/ 33 | 8/ 8 | 4/ 2 | 8/ 11 | n.s. |

BME, bone marrow edema; IKDC, International Knee Documentation Committee; MCID, minimal clinically important difference; SCB, substantial clinical benefit; PASS, patient acceptable symptom state

*^a^* The values are given as number of patients, otherwise noted separately.

*^b^* Group 1, patients without BME; BME 1, patients with a maximum BME grade of 1; BME 2, patients with a maximum BME grade of 2; BME 3, patients with a maximum BME grade of 3.

*^c^* The values are given as the mean and standard deviation.

*^d^* Comparison of the corresponding preoperative values

**Supplementary Table 4** Post-hoc analysis using Scheffé’s method for the variables that were statistically significant in the multiple group comparison

| Variables*^a^* |  | Group 1*^b^* | BME 1*^b^* | BME 2*^b^* | BME 3*^b^* |
| --- | --- | --- | --- | --- | --- |
| Preoperative HKA angle | Group 1 | - | 0.101 | 0.672 | 0.997 |
|  | BME 1 | 0.101 | - | 0.083 | 0.159 |
|  | BME 2 | 0.672 | 0.083 | - | 0.794 |
|  | BME 3 | 0.997 | 0.159 | 0.794 | - |
| Postoperative 2-year HKA angle | Group 1 | - | 0.089 | 0.715 | 0.996 |
|  | BME 1 | 0.089 | - | 0.089 | 0.140 |
|  | BME 2 | 0.715 | 0.089 | - | 0.833 |
|  | BME 3 | 0.996 | 0.140 | 0.833 | - |
| Final follow-up HKA angle | Group 1 | - | 0.269 | 0.422 | 0.935 |
|  | BME 1 | 0.269 | - | 0.070 | 0.195 |
|  | BME 2 | 0.422 | 0.070 | - | 0.710 |
|  | BME 3 | 0.935 | 0.195 | 0.710 | - |

HKA, Hip-Knee-Ankle; BME, bone marrow edema

*^a^* The values are given as p-value derived by comparison of variables of the corresponding subgroups

*^b^* Group 1, patients without BME; BME 1, patients with a maximum BME grade of 1; BME 2, patients with a maximum BME grade of 2; BME 3, patients with a maximum BME grade of 3.
